# Implications of Social Anxiety Symptoms in Adults with Autism Spectrum Disorder: Is There a Predictive Role of Interpersonal Sensitivity and Substance Abuse?

**DOI:** 10.3390/brainsci13111559

**Published:** 2023-11-07

**Authors:** Barbara Carpita, Ivan Mirko Cremone, Benedetta Nardi, Giulia Amatori, Chiara Bonelli, Enrico Massimetti, Danila Casagrande, Stefano Pini, Liliana Dell’Osso

**Affiliations:** 1 Department of Clinical and Experimental Medicine, Section of Psychiatry, University of Pisa, 67 Via Roma, 56126 Pisa, Italybenedetta.nardi@live.it (B.N.); giulia.amatori@libero.it (G.A.); stefano.pini@unipi.it (S.P.); liliana.dellosso@unipi.it (L.D.); 2 North-Western Tuscany Region NHS Local Health Unit, Department of Psychiatry, Head Office, Via Cocchi 7/9, 56121 Pisa, Italy

**Keywords:** autism spectrum disorder, social anxiety disorder, autism, interpersonal sensitivity, substance abuse

## Abstract

Social anxiety disorder (SAD) has been frequently reported by subjects with Autism Spectrum Disorder (ASD). However, interestingly, the overlap between social anxiety and autistic traits may sometimes impede ASD diagnosis in subjects without intellectual or language impairment. The aim of the present work was to evaluate the presence and correlates of social phobic features among subjects with ASD, with a specific focus on evaluating which social anxiety symptoms may be statistically predictive of an ASD diagnosis. With this purpose, 48 subjects with ASD and 48 gender- and age- matched healthy controls (HCs) were recruited and assessed with the SHY-SV and the AdAS Spectrum questionnaires. Results highlighted higher scores in all SHY-SV Spectrum domains and total scores for the ASD group. Moreover, AdAS Spectrum scores were significantly correlated with all SHY-SV domain and total scores. A logistic regression analysis highlighted the SHY-SV *Interpersonal sensitivity* and *Substance Abuse* domains scores as significant positive predictors of an ASD diagnosis. These results confirm the link between ASD and SAD. Because of this association, particular attention should be paid to subjects with high interpersonal sensitivity traits and substance abuse problems.

## 1. Introduction

Autism spectrum disorder (ASD) is a heterogenous neurodevelopmental disorder characterized by difficulties in social interaction and communication, narrow interests, repetitive behaviors, and hypo- or hypersensitivity to sensory stimuli; ASD can present with or without intellectual disability or language development differences [1]. The description and the diagnostic criteria of ASD reported in DSM-5-TR [1] refer mainly to the full-threshold forms but, more recently, ASD symptoms have been conceptualized to lie along a continuum of manifestations, from subtle to severe, and varying greatly in terms of clinical characteristics, severity, and impact on general functioning [1,2]. For these reasons, despite symptoms significantly impairing functioning over the course of their lifetime, many individuals with milder forms, without language or intellectual impairment, may receive a diagnosis only later in life [3]. Moreover, considering the well-known high comorbidity of ASD with other mental conditions [4,5], the diagnosis is often made difficult by the concomitant presence of other kinds of psychiatric symptoms. Indeed, more than 40% of adults with ASD are affected by at least one other mental disorder including anxiety disorders, obsessive compulsive disorder (OCD), affective disorders, psychosis, and post-traumatic stress disorder (PTSD) [6,7,8,9]. Anxiety disorders are the most common co-occurring conditions among subjects on the autism spectrum, affecting a significantly higher percentage of subjects diagnosed with ASD compared to neurotypical peers [10,11,12,13]. Subjects with ASD may manifest both conventional and autism-specific anxiety symptoms, such as worries about routine and atypical fears and phobias [14,15,16]. Moreover, autism and co-occurring anxiety can present in a complex way since the symptoms of the two conditions often interact or even overlap [17]. In particular, social anxiety disorder (SAD) is frequently diagnosed in subjects with ASD, with a prevalence ranging from 13% to 50% when evaluated by trained psychiatrists [6,8,18,19,20,21,22,23,24]. However, because of its similar presentation, it may be challenging to distinguish SAD symptoms from the social avoidance that is frequently described in ASD [25,26].

According to the Diagnostic and Statistical Manual of Mental Disorders, Fifth Edition–Text Revision (DSM-5-TR), SAD is a syndrome characterized by physiological anxiety that appears before or during social settings, and encompasses worries about being poorly perceived and a propensity to avoid interactions [1]. These fears typically cover a wide range of scenarios, such as social interactions (like talking with strangers or meeting new people), being watched (for example, while eating or drinking), and performing in front of others (for example, giving a speech) [1,27,28]. Usually, the disorder has an early onset, arising during adolescence and persisting for several years before professional help is sought, and, because of its chronic course [29], it can often lead to serious functional disability and to a noticeably diminished quality of life [30]. While some patients only have moderate symptoms, others experience symptoms that are widespread and interfere with most social interactions [2,31]. According to epidemiological studies, SAD has a lifetime prevalence rate of 13% in the United States [32] and a similar rate in Europe [33], making it one of the most prevalent mental illnesses worldwide. In addition, comorbid SAD is often present alongside many other psychiatric disorders and, in particular, seems to occur in up to 50% of children diagnosed with ASD, making it one of the most common disorders diagnosed in ASD subjects [34,35,36,37,38]. Unfortunately, there is still limited research on the mechanisms and processes related to this increased comorbidity in school-aged children [37], which is a critical oversight given that the co-occurrence of ASD and SAD may be associated with a diminished treatment response compared to children with only one of the two conditions [38].

As previously stated, several studies have not only reported that many subjects with ASD also meet the criteria for SAD, but have also indicated a significant overlap between symptoms of the two disorders [5,37,39]. ASD and SAD mostly show similar features in the areas of social interaction and social skills [40]; several factors have been hypothesized to be responsible for this convergence. For example, it is possible that some people with ASD or autistic traits eventually experience social anxiety as a result of ongoing struggles in social situations [40,41]. Similarly, in high-functioning ASD, subjects report increased awareness of their communication deficits, alongside low self-perceived social competence; this could contribute to the manifestation of anxious symptoms in social situations [42]. Moreover, the high incidence of social anxiety among biological relatives of subjects with ASD, has led to the suggestion of the existence of some sort of genetic overlap [43,44]. The overlap between social anxiety and autistic traits, along with the possibility of environment-specific anxiety symptoms, not only makes the diagnosis of ASD substantially more difficult, but may also have an impact on potential functional outcomes [44]. Under this framework, it is necessary to recognize the potential presence of significant autistic traits or full-threshold ASD diagnoses underlying diagnoses of SAD in order to guarantee the most appropriate treatment path, thus reducing the impact on quality of life and functional impairment.

Using this framework, the aim of the present work was to evaluate the relationship between social anxiety and autism symptoms in a sample of in- and out-patients diagnosed with ASD compared to gender/age matched healthy controls (HCs), with a specific focus on evaluating which social anxiety symptoms may be more specifically associated with and statistically predictive of an ASD diagnosis. For this purpose, the following two self-report questionnaires were used: the Adult Autism Subthreshold Spectrum Questionnaire (AdAS Spectrum) and the Social Anxiety Spectrum–Short Version (SHY–SV), both validated to assess the typical manifestations of the two disorders as well as less common presentations, temperamental traits, and other additional noteworthy clinical characteristics linked to core symptoms.

Given that the combined presence of ASD and SAD may imply worse treatment outcomes compared to patients with only one condition [45,46,47,48], a better understanding of overlapping features between the two conditions, including patterns of association between specific clusters of symptoms, seems crucial. Shedding light on the connections between the spectra of autism and social anxiety in this context may help to change current prevention and diagnostic strategies, eventually opening the way to new treatment approaches that could improve the quality of life of those affected.

## 2. Materials and Methods

### 2.1. Study Sample and Procedure

The sample was composed of 96 subjects divided into two groups, one of adult patients with ASD (ASD) without language or intellectual impairment, and one of HCs. Each group was composed of 48 subjects. Participants for the ASD group were recruited from in- and out-patients of the Psychiatric Clinic of the University of Pisa. HCs were chosen from a pool of health care and paramedical personnel following sex- and gender-matched criteria. Exclusion criteria were an age under 18 or over 70 years, the inability to provide signed informed consent, language or intellectual disabilities that hindered examination, mental disabilities, lack of collaboration, or persistent psychotic symptoms. HCs subjects were assessed with the Structured Clinical Interview for DSM-5, Research Version (SCID-5–RV) to confirm the lack of mental disorders [49]. The study was conducted in accordance with the Declaration of Helsinki. The study was thoroughly explained to the eligible individuals who then gave their written informed consent after having the opportunity to ask any questions. Subjects did not receive any compensation for taking part in the study. The diagnostic assessment was carried out by trained mental health professionals of the Psychiatry unit of Pisa University Hospital.

### 2.2. Measures

All subjects were assessed with the SCID-5–RV [49]. Participants also completed the SHY–SV and the AdAS Spectrum questionnaires.

#### 2.2.1. The SHY-SV Questionnaire

The SHY–SV is a questionnaire designed by Dell’Osso et al. [50] for the evaluation of the whole spectrum of social anxiety, including common manifestations, atypical symptoms, and personality traits, comprising 139 items organized in 5 domains (Interpersonal sensitivity, Behavioral inhibition, Performance, Social situations, and Substance Abuse) and one appendix (Childhood and adolescence). Each item’s answers are coded in a dichotomous way (yes/no) and the scores relating to the individual domains and appendix are calculated by counting the number of positive answers. In the validation study, carried on a sample of 145 subjects, distributed between three diagnostic groups (SAD, Obsessive compulsive disorders, and HCs), the SHY–SV reported great test-retest reliability, and convergent validity with other dimensional measures of SAD. In particular, the SHY–SV showed strong internal consistency with the overall scale achieving a Cronbach’s alpha of 0.975. Both the total and domain scores had great test-retest reliability with all ICCs above the value of 0.90. The Pearson's coefficients for the SHY–SV domain scores ranged from 0.391 to 0.933, and they were positively and significantly correlated with one another (*p* 0.001) [50]. The questionnaire was developed as a shortened and updated version of the SHY–SR, the first questionnaire specifically designed as a dimensional approach to the social anxiety spectrum, which was proven useful in previous research [51].

#### 2.2.2. AdAS Spectrum

AdAS Spectrum is a self-report questionnaire, developed by Dell’Osso et al. [52], that aims to evaluate, in adults without language or intellectual impairment, the full range of autism spectrum manifestations. It is composed of 160 items classified into the following seven domains: Childhood and adolescence, Verbal communication, Nonverbal communication, Empathy, Inflexibility and adherence to routine, Restricted interests and rumination, and Hyper- and hypo-reactivity to sensory input. The item’s answers are coded in a dichotomous way (yes/no) and the scores relating to the individual domains and appendix are calculated by counting the number of positive answers. The diagnostic threshold is defined as scores of at least 70. In the validation study, the AdAS Spectrum reported a high internal consistency, excellent test-retest reliability, and convergent validity with other dimensional measures of autism spectrum diagnosis [53], with results confirmed by later studies [53,54]. In particular, the AdAS Spectrum demonstrated excellent internal consistency for the total score (Kuder–Richardson's coefficient = 0.964) and sound test-retest reliability (ICC = 0.976). The total and domain AdAS Spectrum scores showed a moderate to strong (>0.50) positive correlation with one another and with the AQ and RAADS-14 total scores [52].

### 2.3. Statistical Analyses

We used Student’s *t*-test and Chi-square tests for the comparison of socio-demographic variables among groups. Student’s *t*-test was also used to comparing the means of AdAS Spectrum and SHY–SV between groups. In order to evaluate the pattern of correlations among scores reported on the two psychometric instruments in the sample, a Pearson’s correlation coefficient was performed. Additionally, a logistic regression analysis was performed with ASD diagnosis as dependent variable and SHY–SV domain scores as independent variables in order to evaluate which SAD symptoms may be statistically predictive of the presence of ASD. All statistical analyses were performed with SPSS version 26.0.

## 3. Results

The ASD sample was composed of 24 (50.0%) males and 24 (50.0%) females with a mean age of 34.02 years (±11.37). The HCs group was composed of 21 (43.8%) males and 27 (56.3%) females with a mean age of 34.15 years (±11.10) (see Table 1).

Table 2 shows the Cronbach’s alphas for the total score of the AdAS Spectrum and the SHY–SV computed for both groups. Both scales demonstrated a high level of internal consistency. Table 3 shows the AdAS Spectrum and SHY–SV scores for the ASD and HC groups. Student’s *t*-test results showed that the ASD group scored significantly higher in all SHY–SV and AdAS Spectrum domains as well as in their total compared to the HC group (see Table 4 and Table 5).

According to the correlational analyses, in the total sample, the total AdAS Spectrum scores and the scores obtained for each domain were significantly and positively correlated with all SHY–SV domains and its total. All correlation coefficients were medium to strong (see Table 6). Table 7 and Table 8 show the correlational analyses for the HC and the ASD groups, respectively. In the HC group, the AdAS domain *Verbal Communication* was significantly and positively correlated with the SHY–SV total score and its domains *Interpersonal Sensitvity*, *Behavioral inhibition,* and *Social situations*. In the ASD group, the AdAS total score and its *Childhood and Adolescence*, *Verbal Communication*, *Non Verbal Communication,* and *Hyper*-/*Hypo-reactivity to sensory input* domains were all positively correlated with the SHY–SV domain, *Interpersonal sensitivity*.

Results from the logistic regression analysis highlighted the SHY–SV *Interpersonal sensitivity* and *Substance Abuse* domains scores as significant positive predictors of an ASD diagnosis (see Table 9).

**Figure 1:**
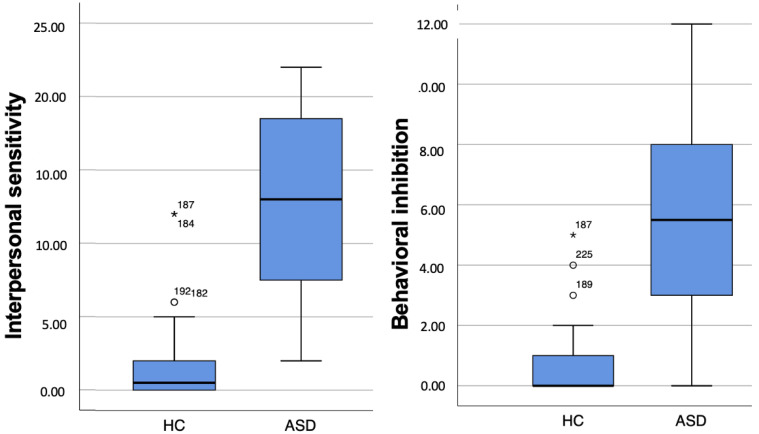

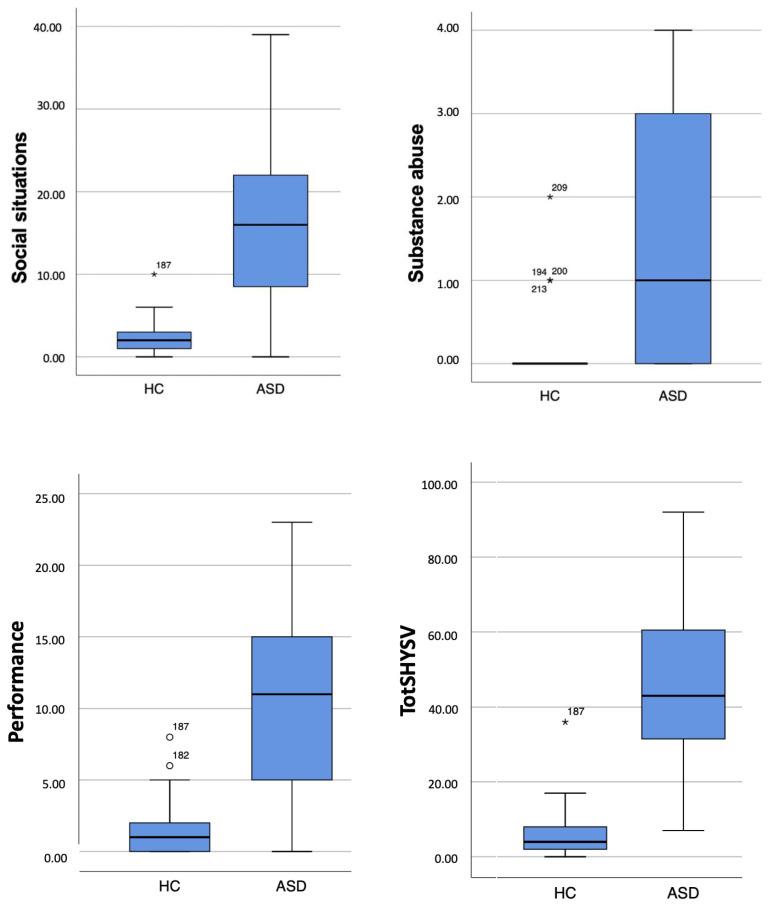
SHY–SV scores boxplots.

**Figure 2:**
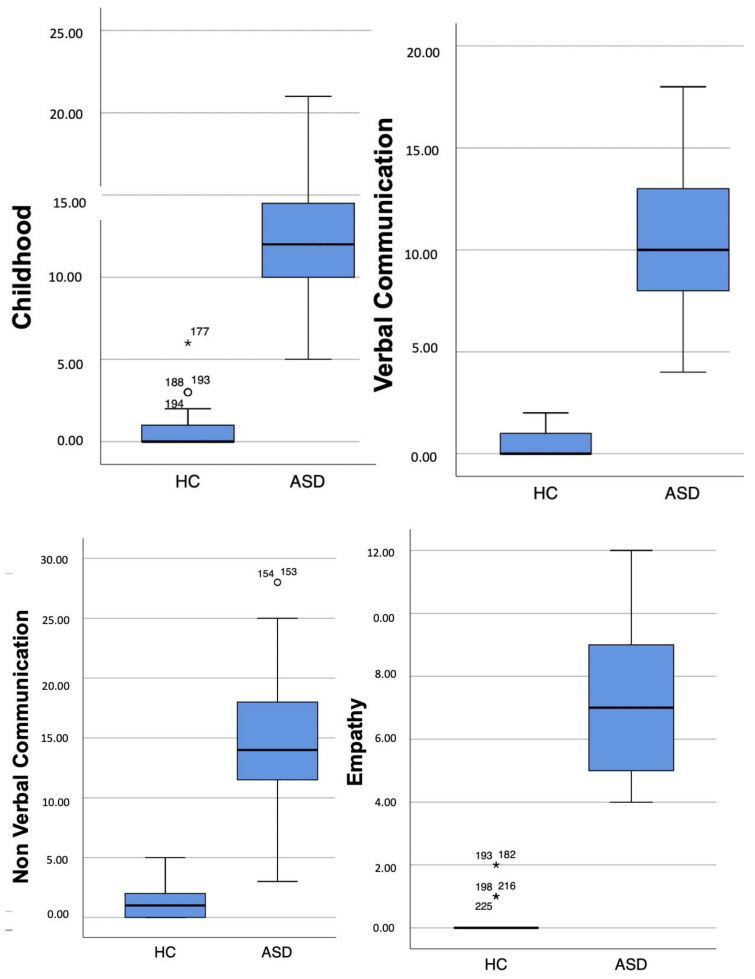

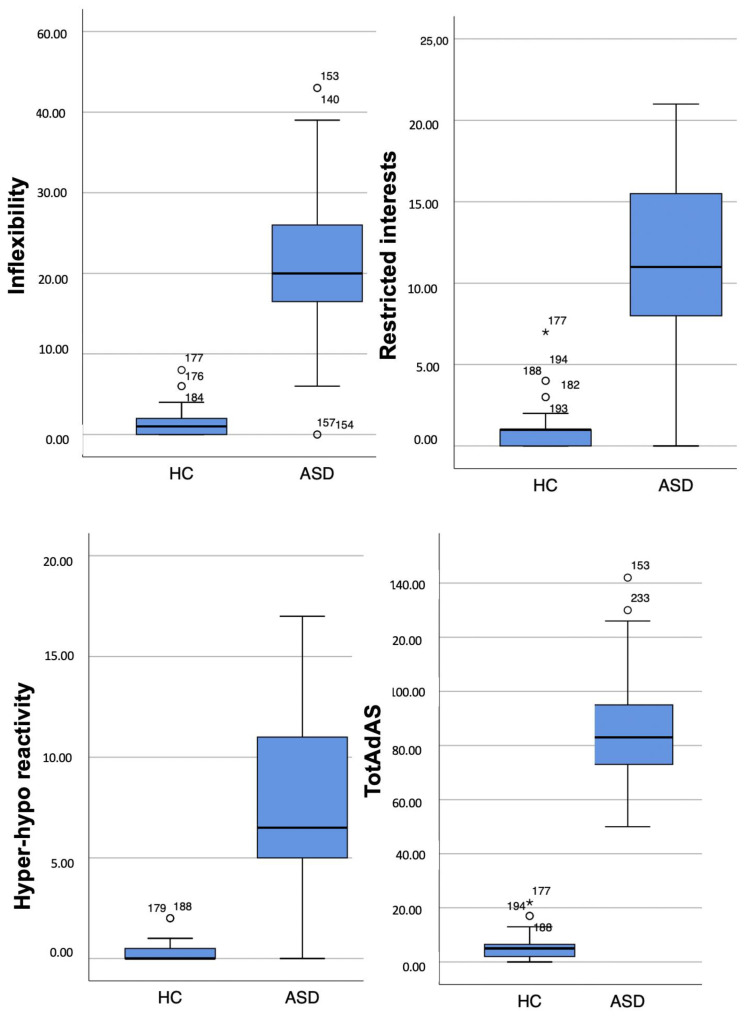
AdAS Spectrum scores boxplots.

Lastly, a boxplot representation of both questionnaires scores is provided in order to verify significant findings were not driven by outliers (see Figure 1 and Figure 2).

## 4. Discussion

Results from our study showed that subjects who received a diagnosis of ASD reported not only significantly higher autistic traits on the AdAS Spectrum questionnaire, but also significantly greater social anxiety traits, as shown by significantly higher scores on the SHY–SV. Our results add to knowledge about the overlap of these symptoms by highlighting a strong significant positive correlation between AdAS Spectrum and SHY–SV scores, in both total scores and single domains (see Table 4). Lastly, to our knowledge, this is the first study to evaluate and report social anxiety features such as Interpersonal sensitivity and Substance Abuse as significant positive predictors of an ASD diagnosis.

Our findings are consistent with most recent literature that investigates the relationship between SAD and ASD. Indeed, our results highlighted the presence of significantly higher SHY–SV scores in the ASD sample compared to HCs, confirming the high prevalence of social anxiety symptoms in the ASD population [6,8,18,19,20,21,22,23,24]. This outcome can be explained by the role that core deficits in social skill acquisition and application, as well as the weak social competence typical of ASD, may show on worsening the disorder’s course, including an increased risk of developing SAD early in life [55,56,57,58]. A larger body of research points to a number of plausible explanations, including deficiencies in social competence, explaining why children with ASD and neurotypical children who exhibit heightened autism traits are more likely to develop SAD [55,56,57,58]. For instance, children and adolescents with ASD are more likely to be bullied or mistreated because of their poor social skills [59], resulting in an abundance of failed interactions with peers [60]. Moreover, ASD subjects, because of the nature of the disorder, which includes deficits in social communication, might be subjected to persistent social rejection and failure that would lead to negative self-perception, especially among subjects with higher levels of insight [14,61], and to an increased tendency to interpret social cues or events negatively or as dangerous [62,63]. Ultimately, these unfavorable interactions and stressful situations frequently serve as early triggers or direct factors leading to the emergence of SAD [62,63,64,65].

In this context, the understanding of the mechanisms and processes by which ASD subjects share a higher risk for co-occurring SAD appears crucial, given that their combined presence predicts worse treatment results compared to subjects presenting with either condition alone [45,46]. Moreover, the correlation between ASD and SAD highlighted in our research is in line with the current literature that explored the relationship between the two disorders both in clinical and non-clinical samples [66,67]. To date, many studies have demonstrated that the symptoms of ASD and SAD significantly overlap in various areas, resulting not only in an increased difficulty in distinguishing between the two disorders [25,26], but also in recognizing the underlying presence of subthreshold autism spectrum traits or even a high functioning ASD among subjects showing social anxiety symptoms. 

According to our results, both interpersonal sensitivity and substance abuse appear to be significant predictors of the presence of autistic traits. These results are consistent with the body of evidence that reports how subjects with ASD are more likely to evaluate themselves as less socially adept compared to their neurotypical peers, and to have more negative self-perception and a lower quality of life [68,69]. In a similar way, patients suffering from SAD are known to experience more unsuccessful social interactions and to perceive themselves as less socially competent compared to typical peers [70,71,72,73,74]. Interestingly, difficulties in social interaction related to impaired theory of mind, the sociocognitive ability to infer others' thoughts that is typically impeded in patients with ASD, were also reported in social anxiety, with the involvement of complex interactions between the central and peripheral network [24,75,76,77,78]. Treatments focused on improving the ability to understand the mental state of others, such as Mentalization-Based Treatment (MBT), have been successfully used in both patients with ASD and individuals with social avoidance [79,80,81]. 

Furthermore, previous studies have proposed that theory of mind might be impaired with the use and abuse of substances, such as alcohol and amphetamines, and its alteration could explain the negative social and interpersonal outcomes observed in the course of these disorders. In addition, brain structures involved in theory of mind have been found to be disrupted under substance use conditions [81]. The use of substances. such as alcohol or opioids, to alleviate the anxiety experienced in social situations has been extensively studied in clinical samples of SAD [82,83,84]; in parallel, the risk of substance abuse has been reported to be double in the ASD population compared to non-clinical populations [85]. Studies suggest these two primary reasons for the misuse of alcohol or illicit substances in individuals with ASD: to fit in with their peers [86] and to self-medicate [87]. In this cases, individuals with ASD use alcohol or other substances to calm sensory stimulation, boost social empathy, facilitate social interaction, soothe stress, and relieve anxiety. 

Considering the results obtained and the information regarding this important association, it is therefore advisable to investigate the presence of relevant autistic traits in patients who show high interpersonal sensitivity traits and substance abuse. On the other hand, it should be taken into account that ASD subjects with social anxiety symptoms may show greater interpersonal sensitivity and altered metacognitive skills, with an increased risk of substance abuse related to complications of SAD. 

**Limitations and Future Directions

Results from this study should be considered in light of some important limitations. First of all, our sample was limited in size, reducing the extensibility and interpretability of our data. In particular, this issue did not allow us to perform more in-depth analyses, which should be carried out in future research using larger samples. Moreover, data for comorbid psychiatric diagnoses or current pharmacological treatment were not available. In addition, we used psychometric questionnaires administered by self-report; this method may open to bias related to under- or over-estimation of symptoms by participants. Finally, the cross-sectional design of the study did not allow us to evaluate possible temporal or causal relationships between the variables under investigation.

Understanding the mechanisms and processes through which ASD participants share greater co-occurring SAD symptoms is essential, given that the combined presence of ASD and SAD may imply a worse outcome. At the same time, identifying possible recurring SAD traits in ASD subjects may improve early identification of milder forms of ASD, as well as of significant autistic traits that may remain under recognized and masked by other psychopathological symptoms in adult patients. Using this framework, our results may open the way to further studies in this field, which may allow for improved prevention and early identification strategies, as well as, eventually, more effective treatment approaches.

## 5. Conclusions

In conclusion, our results confirm the link between ASD and SAD, and describe not only a high prevalence of social anxiety symptoms in ASD subjects but also significant correlations between many core features of the two disorders. These findings support the hypothesis that different psychiatric illnesses may develop as a result of a neurodevelopmental alteration similar to the one associated with ASD [88,89], where the wide range of ASD manifestations can be seen as the tip of a larger iceberg that includes other psychiatric illnesses. In this context, understanding the mechanisms and processes that connects autism and social anxiety spectra may help modify current treatments or develop novel interventions to enhance the quality of life of affected individuals. Moreover, since the comorbidity of ASD and SAD raises the likelihood of developing other clinical issues such anxiety and mood disorders, drug or alcohol abuse, having a lower quality of life [48,90], and a higher risk of suicide [91], the timely treatment of this early-onset problem has the potential to significantly lower the burden of psychiatric illness in later life and reduce the substantial burden of disability.

## Figures and Tables

**Table 1:** Age and sex in the Overall Sample and Comparisons Between Diagnostic Groups.

		ASD	HC		
		Mean ± SD	Mean ± SD	T	*p*
**Age**		34.02 ± 11.37	34.15 ± 11.10	0.054	0.096
		**n (%)**	**n (%)**	**Chi-square**	
**Sex**	**M**	24 (50.0)	21 (43.8)	0.376	539
	**F**	24 (50.0)	27 (56.3)		

**Table 2:** AdAS Spectrum and SHY–SV internal consistency.

Scales	Number of Items	Group	Cronbach’s Alpha
**SHY-SV**	108	HC	0.857
		ASD	0.945
**AdAS Spectrum Total score**	160	HC	0.781
		ASD	0.904

**Table 3:** AdAS Spectrum and SHY–SV scores for the ASD and HC groups.

Group			AdAS Spectrum	SHY-SV
**HC**	Mean		5.31	5.75
	Median		5.00	4.00
	Mode		5.00	4.00
	St. deviation		4.75	6.01
	Minimun		0	0
	Maximum		22	36
	Percentiles	25	2.00	2.00
		50	5.00	4.00
		75	6.75	8.00
**ASD**	Mean		85.79	46.10
	Median		83.00	43.00
	Mode		82.00	25.00
	St. deviation		19.37	19.66
	Minimun		50	7
	Maximum		142	92
	Percentiles	25	73.00	31.25
		50	83.00	43.00
		75	95.50	61.25

Note: For the AdAS Spectrum, 43 marks indicate significant autistic traits, and 70 marks indicate clinically significant ASD symptoms.

**Table 4:** Comparison of SHY–SV scores between the diagnostic groups.

SHY-SV Scores	ASD Group Mean ± SD	HC Group Mean ± SD	t	*p*
**Interpersonal sensitivity**	12.62 ± 6.37	1.52 ± 2.67	−11.41	<0.001
**Behavioral inhibition**	5.81 ± 3.38	0.62 ± 1.10	−10.10	<0.001
**Social situations**	15.83 ± 9.48	2.14 ± 1.99	−9.79	<0.001
**Substance abuse**	1.50 ± 1.43	0.23 ± 0.47	−5.85	<0.001
**Performance**	10.33 ± 6.20	1.23 ± 1.63	−9.84	<0.001
**Total**	46.10 ± 19.66	5.75 ± 6.01	−13.60	<0.001

**Table 5:** Comparison of AdAS Spectrum scores between the diagnostic groups.

AdAS Spectrum Scores	ASD Group Mean ± SD	HC Group Mean ± SD	t	*p*
**Childhood/Adolescence**	12.69 ± 4.01	0.83 ± 1.21	−19.60	<0.001
**Verbal communication**	10.52 ± 3.61	0.48 ± 0.62	−19.00	<0.001
**Non-verbal communication**	14.39 ± 5.92	1.04 ± 1.34	−15.25	<0.001
**Empathy**	7.46 ± 2.53	0.21 ± 0.50	−19.44	<0.001
**Inflexibility & adherence to routine**	20.85 ± 9.87	1.54 ± 1.75	−13.35	<0.001
**Restricted interest & rumination**	11.87 ± 5.03	0.89 ± 1.36	−14.59	<0.001
**Hyper-/hypo-reactivity to sensory input**	8.00 ± 5.00	0.31 ± 0.59	−10.57	<0.001
**Total Score**	85.79 ± 19.37	5.31 ± 4.75	−27.96	<0.001

**Table 6:** Pearson’s correlation coefficients (r) between AdAS Spectrum domains score and SHY–SV scores in the total sample.

	Interpers. Sens.	Behav. Inhib.	Social Sit.	Subst. Abuse	Performance	Tot. Score
**Child./Adolesc.**	0.778 *	0.627 *	0.607 *	0.440 *	0.560 *	0.724 *
**Verb. comm.**	0.792 *	0.726 *	0.638 *	0.518 *	0.643 *	0.780 *
**Non-verb. comm.**	0.784 *	0.617 *	0.626 *	0.478 *	0.568 *	0.736 *
**Empathy**	0.740 *	0.652 *	0.609 *	0.510 *	0.655 *	0.745 *
**Inflex. & routine**	0.563 *	0.603 *	0.646 *	0.401 *	0.662 *	0.696 *
**Restrict. interest & rum.**	0.626 *	0.629 *	0.664 *	0.452 *	0.649 *	0.725 *
**Hyper-hyporeact.**	0.702 *	0.579 *	0.497 *	0.377 *	0.458 *	0.622 *
**Tot. Score**	0.782 *	0.707 *	0.698 *	0.503 *	0.682 *	0.807 *

* Significant correlation with *p* < 0.001, threshold for statistical significance according to Bonferroni correction in this group (0.005/48).

**Table 7:** Pearson’s correlation coefficients (r) between AdAS Spectrum domains score and SHY–SV scores for the HC group.

	Interpers. Sens.	Behav. Inhib.	Social Sit.	Subst. Abuse	Performance	Tot. Score
**Child./Adolesc.**	0.093	−0.191	0.046	−0.081	0.009	0.018
**Verb. comm.**	0.373 *	0.331 *	0.288 *	0.199	0.185	0.388 *
**Non-verb. comm.**	0.149	0.170	0.142	0.119	−0.093	0.129
**Empathy**	0.234	−0.010	0.033	−0.026	0.174	0.158
**Inflex. & routine**	0.139	−0.003	0.087	−0.205	−0.089	0.050
**Restrict. interest & rum.**	0.174	−0.083	0.037	0.005	0.040	0.085
**Hyper-hyporeact.**	0.111	−0.143	0.251	−0.033	−0.010	0.101
**Tot. Score**	0.253	−0.002	0.166	−0.042	−0.004	0.163

* Significant correlation for *p* < 0.05.

**Table 8:** Pearson’s correlation coefficients (r) between AdAS Spectrum domains score and SHY–SV scores for the ASD group.

	Interpers. Sens.	Behav. Inhib.	Social Sit.	Subst. Abuse	Performance	Tot. Score
**Child./Adolesc.**	0.386 *	−0.053	−0.105	−0.058	−0.273	−0.025
**Verb. comm.**	0.416 *	0.265	0.006	0.146	0.021	0.200
**Non-verb. comm.**	0.451 *	0.012	0.068	0.089	−0.088	0.159
**Empathy**	0.226	0.023	−0.090	0.135	0.050	0.059
**Inflex. & routine**	−0.146	0.050	0.174	−0.025	0.222	0.113
**Restrict. interest & rum.**	−0.027	0.086	0.195	0.050	0.150	0.151
**Hyper-hyporeact.**	0.353 *	0.112	−0.065	−0.006	−0.146	0.056
**Tot. Score**	0.334 *	0.122	0.111	0.059	0.041	0.200

* Significant correlation for *p* < 0.05.

**Table 9:** Logistic regression analysis with ASD diagnosis as dependent variable and SHY–SV domain scores as independent variables.

	b (S.E)	*p*	Exp(B)	CI 95%	
				Lower Bound	Upper Bound
constant	−5.44 (1.29)	<0.001	0.004		
*Interpers. sens.*	0.29 (0.10)	0.006	1.330	1.09	1.63
*Behav. inhib.*	0.36 (0.34)	0.320	1.430	0.72	2.82
*Social sit.*	0.34 (0.19)	0.072	1.402	0.97	2.02
*Subst. abuse*	1.49 (0.64)	0.020	4.444	1.26	15.67
*Performance*	0.07 (0.18)	0.679	1.076	0.76	1.52

Cox &S nell R square = 0.678; Nagelkerke R square = 0.904. Overall percentage correct: 95.8%.

## Data Availability

The raw data supporting the conclusions of this article will be made available by the authors, without undue reservation.
